#  Characteristic phenotype of Chinese patients with adult-onset diabetes who are autoantibody positive by 3-Screen ICA™ ELISA

**DOI:** 10.1007/s00592-021-01778-8

**Published:** 2021-09-17

**Authors:** Zhida Wang, Liang Guo, Shu Chen, Jun Guan, Michael Powell, Jadwiga Furmaniak, Bernard Rees Smith, Liming Chen

**Affiliations:** 1 NHC Key Laboratory of Hormones and Development, Tianjin Key Laboratory of Metabolic Diseases, Tianjin Medical University Chu Hsien-I Memorial Hospital & Tianjin Institute of Endocrinology, Tianjin Medical University, Tianjin, China; 2 FIRS Laboratories, RSR Ltd Parc Ty Glas, Llanishen, Cardiff, CF14 5DU UK; 3 RSR Tianjin Biotech Ltd, Haitai Green Area, Hi Huayuan Industrial Park, Tianjin, 300384 China

**Keywords:** T2DM, Diabetes autoantibodies, GADAb, IA-2Ab, ZnT8Ab, β-cell function

## Abstract

**Aims:**

To assess the prevalence of diabetes-associated autoantibodies in Chinese patients recently diagnosed with adult-onset diabetes and to evaluate the potential role of the autoantibody markers for characterization of disease phenotype in the patient population.

**Methods:**

The study included 1273 recent-onset adult patients with phenotypic type 2 diabetes mellitus (T2DM). Serum samples were tested using the 3-Screen ICA™ ELISA (3-Screen) designed for combined measurement of GADAb and/or IA-2Ab and/or ZnT8Ab. 3-Screen positive samples were then tested for individual diabetes-associated and other organ-specific autoantibodies. Clinical characteristics of patients positive and negative in 3-Screen were analysed.

**Results:**

Forty-four (3.5%) of the T2DM patients were positive in 3-Screen, and 38 (86%) of these were also positive for at least one of GADAb, IA-2Ab and ZnT8Ab in assays for the individual autoantibodies. 3-Screen positive patients had lower BMI, higher HbA1c, lower fasting insulin levels and lower fasting C-peptide levels compared to 3-Screen negative patients. Analysis using a homeostatic model assessment (HOMA2) indicated that HOMA2-β-cell function was significantly lower for the forty-four 3-Screen positive patients compared to 3-Screen negative patients.

Twenty (45%) 3-Screen positive patients were also positive for at least one thyroid autoantibody.

**Conclusions:**

The 3-Screen ELISA has been used successfully for the first time in China to detect diabetes autoantibodies in patients with phenotypic T2DM. 3-Screen positive patients presented with poorer β cell function.

## Introduction

Presentation of type 2 diabetes mellitus (T2DM) is typically heterogeneous in different patients [1, 2] with 4–10% of adults clinically diagnosed with T2DM presenting with a phenotype similar to that of type 1 diabetes mellitus (T1DM) and with serological markers of autoimmune β-cell damage [2–5]. Autoantibodies (Ab) to glutamic decarboxylase (GAD), insulinoma-associated antigen 2 (IA-2), zinc transporter 8 (ZnT8) and insulin are used widely to identify β-cell autoimmunity in diabetes [6–8].

This study aimed to assess the prevalence of diabetes-associated autoantibodies in a cohort of Chinese patients recently diagnosed with adult onset T2DM using the new 3-Screen ICA™ ELISA (3-Screen) for the combined measurement of GADAb, IA-2Ab and ZnT8Ab in a single serum sample. Further, we aimed to evaluate the potential value of the autoantibody markers for characterization of a disease phenotype in particular, the clinical characteristics and metabolic profiles of 3-Screen positive patients compared to 3-Screen negative patients. Recognition of the characteristic disease phenotype may be helpful in tailoring treatment regimens specifically for 3-Screen positive Chinese adult T2DM patients.

## Materials and methods

### Patients

A hospital-based cross-sectional study was conducted in Tianjin Chu Hsien-I Memorial Hospital, Tianjin, China. A total of 1383 adult patients with recently diagnosed diabetes mellitus (less than one year) were recruited between April 2014 to October 2016. Diabetes mellitus was diagnosed using the criteria described by Sacks et al. [10] and the recommendations of the American Diabetes Association [11]. This included assessment based on clinical presentation, clinical history, oral glucose tolerance test and insulin releasing test according to the Chinese guidelines for the prevention and treatment of T2DM [12]. In all 1273 patients with phenotypic T2DM diagnosis were enrolled in the study using specific inclusion criteria: (a) diagnosis of diabetes based on fasting blood sugar (FBS) higher than 7.0 mmol/L and postprandial and random blood sugar higher than 11.1 mmol/L in patients aged ≥ 18yrs; (b) disease duration < 1 year; and (c) no diabetic ketoacidosis (DKA) in the first 6 months after the diagnosis of diabetes.

Patients (*n* = 110) with: (a) gestational diabetes, (b) secondary diabetes and/or with concurrent malignancies and (c) acute-onset type 1 diabetes or fulminant type 1 diabetes were excluded.

Age, sex, height, weight, body mass index (BMI), waist circumferences, hip circumferences and systolic and diastolic blood pressure were recorded at the time of entry into the study. Furthermore, disease-associated parameters including glycated haemoglobin A1c levels (HbA1c), fasting blood sugar, fasting C-peptide and insulin were tested using standard methods at the biochemistry laboratories in Tianjin Chu Hsien-I Memorial Hospital. The combined impact of these measurements was assessed using a Homeostasis Model Assessment (HOMA2, www.dtu.ox.ac.uk/homa) to derive estimates of β-cell function (% B) and insulin sensitivity (S%).

At enrolment all 1273 patients classed as phenotypic T2DM were assigned to various treatment regimens including oral diabetes medication or insulin therapy or combination of both if the patient could not maintain the target blood sugar level with diet and exercise.

### Autoantibody assays

Serum separated from fasting venous blood sample was stored at -80 °C. Combined measurements of GADAb, IA-2Ab and ZnT8Ab were carried out using the recently developed multiplex 3-Screen ICA™ ELISA (RSR Ltd., Cardiff, UK) [13]. Briefly, sera (25 µL singly) were incubated (overnight; 2–8 °C) in ELISA plate wells coated with GAD, IA-2 and ZnT8 followed by a wash step and incubation (1 h; 2–8 °C) with biotinylated GAD, IA-2 and ZnT8. The signal was developed by the addition of streptavidin peroxidase (20 min at room temperature) followed by a wash step, addition of tetramethylbenzidine and after 20 min, stop solution. The optical density (OD) of the plate wells was measured at 450 nm and 405 nm using an ELISA plate reader. 3-Screen calibrators (5, 15, 100, 400 and 2000 units/mL; RSR arbitrary units) were included in each assay, and samples with OD values above the 2000 units/mL calibrator were defined as greater than 2000 units/mL. The cut-off value for antibody positivity in the 3-Screen assay (10.0 units/mL) was determined as the 95th percentile of 2534 healthy blood donor (HBD) sera (purchased from Golden West Biological Vista, CA, USA). All patients sera positive in 3-Screen (i.e. > 10 units/mL) were tested for GADAb, IA-2Ab and ZnT8Ab in individual ELISAs [14–16] using kits from RSR Ltd. Values of GADAb ≥ 5.0 units/mL (NIBSC 97/550 units), IA-2Ab ≥ 7.5 units/mL (NIBSC 97/550 units) or ZnT8Ab ≥ 15 units/mL (RSR arbitrary units) were considered positive. These cut-off values were as recommended in the respective kit instructions. Using these cut-off values in the Islet Autoantibody Standardization Program 2016 (IASP 2016), 3-Screen ELISA scored 94% sensitivity and 100% specificity, the GADAb ELISA 76% sensitivity and 99% specificity, IA-2Ab ELISA 76% sensitivity and 98% specificity and ZnT8Ab ELISA 72% sensitivity and 99% specificity [17–19].

All 3-Screen positive samples were also tested for other organ-specific autoantibodies, including: thyroid-stimulating hormone receptor (TSHR) Ab, thyroid peroxidase (TPO) Ab, thyroglobulin (Tg) Ab and steroid 21-hydroxylase (21-OH) Ab. These Abs were measured using ELISA kits from RSR Ltd and following the manufacturer's recommendations, values of TSHRAb > 1.5 units/mL (NIBSC 90/672, second generation TRAb ELISA), TPOAb ≥ 10 units/mL (NIBSC 66/387) and TgAb ≥ 65 units/mL (NIBSC 65/093) were considered positive. Serum 21-OHAb levels ≥ 0.4 units/mL (RSR arbitrary units) were considered positive.

### Statistical analysis

Statistical analysis was performed using GraphPad Prism version 6.07 (GraphPad Software Inc, San Diego, USA). Most of the data were not normally distributed and were expressed as median (interquartile range; IQR). Frequency differences were compared using Chi-square test or Fisher exact test when appropriate. Nonparametric Mann–Whitney U tests were used to compare the numerical data in any two groups. The adjusted *P* value (i.e. false discovery rate *P* value) was calculated using Benjamini–Hochberg multiple testing procedure [20]. For all results, statistical significance was defined by *P* or adjusted *P* < 0.05.

## Results

### Prevalence of diabetes-associated autoantibodies in T2DM patients

**Fig. 1:**
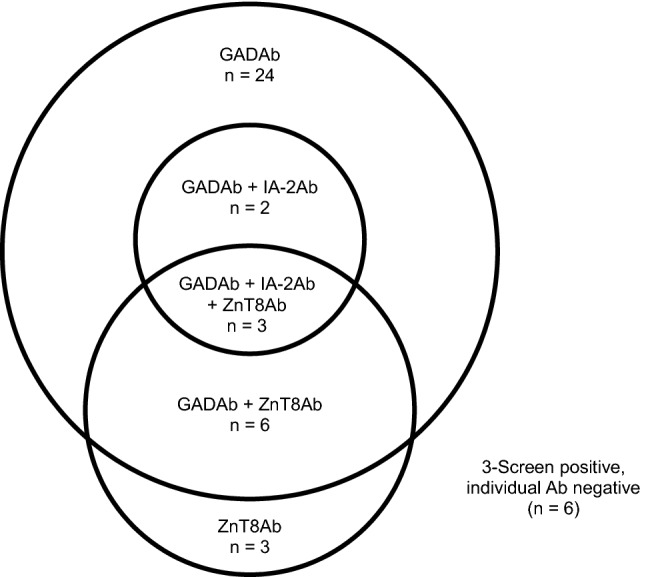
Venn diagram of GADAb, IA-2Ab and ZnT8Ab positivity in 44 patients positive (> 10 units/mL) in 3-Screen ICA™ ELISA

Out of 1273 T2DM patients studied, 44 (3.5%) were 3-Screen positive (> 10 units/mL). In detail, 7 (0.55%) sera were between 10 and 20 units/mL; 14 (1.1%) between 20 and 100 units/mL; 11 (0.86%) between 100 and 2000 units/mL; and 12 (0.94%) greater than 2000 units/mL. In this group of 44 3-Screen positive patients, 35 (80%) were positive for GADAb, 5 (11%) for IA-2Ab, and 12 (27%) for ZnT8Ab in the individual autoantibody assays. Thirty-eight (86%) sera were positive for at least one of GADAb, IA-2Ab and ZnT8Ab, while six 3-Screen positive samples were found negative in the individual ELISAs. Eleven (25%) of the 44 patients had at least two detectable autoantibodies, and the median value in 3-Screen for these 11 patients was greater than 2000 units/mL (ranged from 49 to greater than 2000 units/mL). For 27 (61%) patients who were positive for one autoantibody only (24 for GADAb only and 3 for ZnT8Ab only), the median 3-Screen value was 77.2 units/mL (ranged from 13.2 to greater than 2000 units/mL). These values can be compared to a median 3-Screen level of 16.1 units/mL (ranged from 11.3 to 23.6 units/mL) for the 6 (14%) patients who were negative for all three antibodies when tested in the individual autoantibody ELISAs (Fig. 1).

### Age distribution of 3-Screen positive patients

In the 1273 patients studied, 36 (2.8%) were younger than 30 years old (range 17 to < 30 years old) of which 4/36 (11.1%) were positive in 3-Screen. In the 143/1273 (11.2%) aged from 30 to < 40 years old, 7/143 (4.9%) were 3-Screen positive. In the 186/1273 (14.6%) of patients aged from 40 to < 50 years, 8/186 (4.3%) were 3-Screen positive. In the case of 481/1273 (37.8%) of patients between 50 and < 60 years old, 14/481 (2.9%) were 3-Screen positive, and out of the 427 (33.5%) of patients aged 60 years and over, 11/427 (2.6%) were 3-Screen positive. 3-Screen positivity in different age groups of patients was compared using Chi-square test, and there was no statistically significant difference between any of the groups (*P* = 0.06).

### Clinical features in different patient groups

Tables 1 and 2 show the comparison of the clinical features of 3-Screen positive and negative patients together with statistical significance values for the difference between the two groups in each case. The number of patients who had diabetic ketoacidosis (DKA) and/or required insulin treatment was significantly higher in the 3-Screen positive group than in the 3-Screen negative group (37% vs 17%, respectively, for patients with DKA, *P* < 0.05 and 43% vs 25%, respectively, for patients on insulin treatment, *P* < 0.05). 3-Screen positive patients had significantly lower BMI (24.5 vs 26.0 kg/m^2^, *P* < 0.05), significantly higher HbA1c (9.2 vs 8.0% (77 vs 64 mmol/mol), *P* < 0.05), significantly lower fasting insulin levels (11.6 vs 14.4 mIU/L, *P* < 0.05) and significantly lower fasting C-peptide levels (1.9 vs 2.4 ng/mL, *P* < 0.05) compared to the 3-Screen negative patients (Tables 1 and 2). In a homeostatic model HOMA2 assessment, median HOMA2-β-cell function was significantly lower (36.7 vs 51.9%, *P* < 0.05) for the 44 3-Screen positive patients compared to 3-Screen negative patients. However, HOMA2 insulin sensitivity and insulin resistance were not significantly different for the 3-Screen positive patients compared to 3-Screen negative patients (Tables 1 and 2). There were no statistical differences in terms of patients’ age, sex, duration of diabetes at sample collection, ratio of waist circumferences to hip circumferences, systolic and diastolic blood pressure or fasting blood glucose between 3-Screen positive and negative patients (Tables 1 and 2). The adjusted *P* value in Benjamini–Hochberg multiple test was statistically significant for 4 out 7 variables found significantly different in nonparametric test including the number of patients who had DKA, HbA1c, HOMA2 β-cell function and fasting serum C-peptide (adjusted *P* < 0.05, Tables 1 and 2).

**Table 1:** Clinical features of 3-Screen positive and negative patients with phenotypic type 2 diabetes mellitus (analysed using numeric data)

	3-Screen positive (*n* = 44)	3-Screen negative (*n* = 1229)	Significance of difference	
			*P*	Adjusted *P*
Age (years)	54 (39–60)	56 (48–61)	0.07	0.11
Duration of diabetes at sample collection (months)	2.0 (0.7–3.0)	2.0 (1.0–4.0)	0.25	0.27
BMI (kg/m^2^)	24.5 (22.9–26.8)	26.0 (23.9–28.7)	0.03*	0.07
Waist-to-hip ratio	0.91 (0.87–0.96)	0.92 (0.89–0.95)	0.20	0.23
Systolic BP (mm Hg)	125 (120–140)	130 (120–140)	0.10	0.13
Diastolic BP (mm Hg)	80 (80–90)	80 (80–90)	0.11	0.14
%HbA1c / mmol/mol HbA1c	9.2 (7.6–11.0)/ 77 (60–97)	8.0 (7.0–9.6)/ 64 (53–81)	0.0007*	0.008**
Fasting blood glucose (mmol/L)	9.2 (7.6–12.2)	7.3 (4.8–8.6)	0.10	0.13
Fasting serum C-peptide (ng/mL)	1.9 (1.5–2.9)	2.4 (1.8–3.1)	0.01*	0.04**
Fasting serum insulin (mIU/L)	11.6 (7.3–18.1)	14.4 (9.2–21.9)	0.03*	0.07
HOMA2 β-cell function B%	36.7 (20–79.4)	51.9 (33.3–80.1)	0.006*	0.03**
HOMA2 insulin sensitivity S%	57.6 (37–83.8)	47.7 (31.2–72.8)	0.07	0.11
HOMA2 insulin resistance IR	1.8 (1.2–2.7)	2.1 (1.4–3.2)	0.07	0.11

The data did not have a normal distribution and were expressed as median values with interquartile ranges (IQR) in brackets and were compared by a nonparametric Mann–Whitney U test. *indicates that there was a significant difference (*P* < 0.05) between the two groups. The adjusted *P* value was calculated using Benjamini–Hochberg multiple testing procedure. **indicates that there was a significant difference (adjusted *P* < 0.05) between the two groups

**Table 2:** Clinical features of 3-Screen positive and negative patients with phenotypic type 2 diabetes mellitus (analysed using categorical data)

	3-Screen positive (*n* = 44)	3-Screen negative (*n* = 1229)	Significance of difference	
	Number of patients and percentage (%)		*P*	Adjusted *P*
Number of females (% of total)	14 (32%)	479 (39%)	0.43	0.43
Number of patients with DKA (% of total)	17 (37%)	213 (17%)	0.001*	0.008**
Number of patients on insulin treatment at sample collection (% of total)	18 (43%)	302 (25%)	0.02*	0.06

The difference in the percentage for the respective variables between two patients' groups was compared using Fisher's exact test. *indicates that there was a significant difference (*P* < 0.05) between the two groups. The adjusted *P* value was calculated using Benjamini–Hochberg multiple testing procedure. **indicates that there was a significant difference (adjusted *P* < 0.05) between the two groups

There were no statistically significant differences between 3-Screen positive patients with DKA and/or insulin treatment (*n* = 21) and 3-Screen positive patients without DKA and insulin treatment (*n* = 23) in terms of the clinical features studied except that patients with DKA and/or insulin treatment were much younger (median age of 41 vs 58 years, *P* < 0.05) and had lower systolic blood pressure (Table 3). In addition, 3-Screen Ab levels in patients with DKA and/or insulin treatment were significantly higher than in patients without DKA and insulin treatment (median 717 vs 52 units/mL respectively, *P* < 0.05), and there were more patients’ samples positive for GADAb or IA-2Ab or multiple islet autoantibodies (Table 3 and 4). Six patients positive in 3-Screen but negative in the individual ELISAs had similar clinical features to the 1229 3-Screen negative patients in terms of their BMI of 26.6 (24.9–31.2) kg/m2, HbA1c 8.4 (7.0–9.7) % (68 (53–83) mmol/mol), fasting insulin level 16.9 (10.0–21.0) mIU/L, fasting C-peptide level 2.8 (1.9–3.8) ng/mL and HOMA2-β-cell function 58.4 (23.2–105) % (Table 1).

**Table 3:** Features of 3-Screen positive patients with or without DKA and/or insulin treatment (analysed using numeric data)

	All 3-Screen positive patients (*n* = 44)	3-Screen positive patients without DKA and insulin treatment (*n* = 23)	3-Screen positive patients with DKA and/or insulin treatment (*n* = 21)	Significance of difference *(P)*
Median value and range (IQR)				
Age (years)	54 (39–60)	58 (49–62)	41 (33–58)	0.01*
Systolic BP (mm Hg)	125 (120–140)	130 (120–140)	120 (118–126)	0.006*
3-Screen Ab level (units/mL)	134 (34 ≥ 2000)	52 (18–154)	717 (92 ≥ 2000)	0.006*

The data did not have a normal distribution and were expressed as median values with interquartile ranges (IQR) and were compared by a nonparametric Mann–Whitney U test. *indicates that there was a significant difference (P < 0.05) between the two groups

**Table 4:** Features of 3-Screen positive patients with or without DKA and/or insulin treatment (analysed using categorical data)

	All 3-Screen positive patients (*n* = 44)	3-Screen positive patients without DKA and insulin treatment (*n* = 23)	3-Screen positive patients with DKA and/or insulin treatment (*n* = 21)	Significance of difference *(P)*
Number of patients and percentage (%)				
Number GADAb positive	35 (80%)	15 (65%)	20 (95%)	0.01*
Number IA-2Ab positive	5 (11%)	0 (0%)	5 (24%)	0.01*
Number ZnT8Ab positive	12 (27%)	4 (17%)	8 (38%)	0.12
Number with at least 2 diabetes Ab positive	11 (25%)	1 (4%)	10 (48%)	0.0009*
Number with at least 1 diabetes Ab positive	38 (86%)	18 (78%)	20 (95%)	0.10
Number with at least 1 thyroid Ab positive	20 (46%)	10 (43%)	10 (48%)	0.55

The difference in the percentage for the respective variables between two patients' groups was compared using Fisher's exact test. *indicates that there was a significant difference (P < 0.05) between the two groups

### Other autoantibodies

Non-diabetes autoantibodies were detected in 20/44 (45%) 3-Screen positive patients who were positive for at least one thyroid autoantibody (TPO Ab and/or Tg Ab and/or TSHR Ab), while none were positive for the adrenal autoantibody 21-OHAb. In the case of the 20 thyroid autoantibody positive patients, 4 had hypothyroidism coincident with diabetes with raised TSH levels (6.8—23.8 mIU/L) and lower or normal fT4 levels of 9.4 -13.5 pmol/L. One had a TSH level below the normal range (0.026 mIU/L) and normal fT4 (19.7 pmol/L); however, this sample was TSHR Ab negative. There were no statistically significant differences between thyroid autoantibody positive and negative patients in terms of clinical features analysed except for median systolic blood pressure which was significantly higher (130 vs 120 mm Hg, *P* < *0.05)* in thyroid autoantibody positive patients.

## Discussion

Assays for individual islet cell autoantibodies including GADAb, IA-2Ab and ZnT8Ab are sensitive, specific and standardized [17–19] but are not designed for screening large numbers of samples due to cost and assay time. 3-Screen is a novel ELISA for the combined measurement of Abs to GAD, to IA-2 and to ZnT8 in a single serum sample, and there is good agreement between ELISA results for individual autoantibodies and 3-Screen [13]. 3-Screen has been found to be an excellent tool for screening populations to predict T1DM development. For example, in the T1DM Fr1da study 90,632 Bavarian children were screened initially with 3-Screen and 280 (0.31%) were found positive for multiple β-cell autoantibodies [21].

In this study, we used 3-Screen to identify a specific disease phenotype associated with the presence of β-cell autoantibodies in a relatively large number (*n* = 1273) of Chinese patients with adult onset T2DM. Forty-four (3.5%) patients were positive in 3-Screen and consequently classed as LADA. This can be compared with 5.9% of patients with adult onset diabetes reported to be positive for GADAb in a large multicentre Chinese national study [5]. Furthermore, the ADOPT study reported GADAb positivity of 4.7% in recently diagnosed T2DM patients in North America and 3.7% in Europe [3].

We found no statistically significant differences for 3-Screen positivity in the different age groups of our patients. There was, however, a trend for lower positivity as age increased with 11% of patients below the age of 30 years 3-Screen positive, 4.6% of patients between 30 and 50 years of age and 2.8% of patients older than 50 years positive. Such a trend was also reported in the UKPDS study where 35% of newly diagnosed T2DM patients aged 25–34 years had islet cell antibody (ICA) and/or GADAb compared with 9% in the 55 to 65-year age group [2]. In contrast, in the LADA China study 5, GADAb, IA-2Ab, ZnT8Ab and insulin Ab positivity did not show significant variations with age in a population of T2DM patients aged from 30 years to over 60 years [8].

The majority (80%) of 3-Screen positive patients in our study were positive for GADAb, 27% of patients positive for ZnT8Ab and 11% positive for IA-2Ab in the respective individual autoantibody assays. These results are in agreement with previously reported prevalence of GADAb of 96% in patients with adult-onset autoimmune diabetes compared to ZnT8Ab and IA-2Ab of 22.1% and 19.6%, respectively [7, 22]. Although the majority of our 3-Screen positive (i.e. LADA) patients were positive for GADAb, three patients (7%) were positive for ZnT8Ab only. Therefore, ZnT8Ab may have an additive value to GADAb in identifying LADA patients. This is in agreement with 90% of LADA patients reported positive for GADAb, while 10% were positive for IA-2Ab and/or ZnT8Ab only in the European Action LADA study [23]. Similarly, 96% of LADA patients had detectable GADAb while 4% had IA-2Ab and/or ZnT8Ab only in the Italian NIRAD study [7]. In contrast, a higher proportion of LADA patients (33%) had insulin autoantibodies and/or IA-2Ab and /or ZnT8Ab rather than GADAb in the LADA China Study 5 [8].

Out of the 44 3- Screen positive samples, 38 were positive in at least one individual autoantibody ELISA. In the case of the six 3-Screen only positive samples, 4/6 had levels of GADAb in the ELISA just below the assay cut-off (4.0–4.7 units/mL). Such low concentrations of GADAb as observed in these 4 patients may produce an enhanced GADAb signal under the 3-Screen assay conditions. This is most likely related to differences in the assay protocols for the GADAb ELISA compared to 3-Screen ELISA. 3-Screen test samples are incubated in antigen-coated plate wells overnight at 4 °C while just for 1 h at room temp in the GADAb assay. The longer incubation time may well favour higher binding of low concentrations of GADAb to GAD coated on the 3-Screen ELISA plates. In addition, increased assay sensitivity with the same specificity has been reported for the GADAb ELISA for T1DM patient sera with longer incubation times [24]. Another reason could be an additive signal in 3-Screen from individual antibodies present at low concentrations which were below the cut-off in the individual autoantibody assays.

This study aimed to assess the differences in requirement for insulin treatment and HOMA2 and/or HbA1c in patients with or without diabetes autoantibodies. Compared to 3-Screen negative patients (*n* = 1229), the positive patients (*n* = 44) had lower BMI, higher HbA1c, lower fasting insulin level, lower fasting C-peptide level, lower HOMA2-β-cell function and higher prevalence of DKA and/or insulin treatment, all consistent with typical LADA characteristics (Tables 1, 2).

Furthermore, 3-Screen positive patients who had DKA and/or required insulin treatment (*n* = 22) were younger had higher 3-Screen Ab levels, higher positivity for GADAb, for IA-2Ab and for multiple islet autoantibodies compared to patients without DKA and insulin treatment. However, there were no significant differences in β-cell function assessment using HOMA2 between 3-Screen positive and negative patients (Tables 3 and 4). It has been shown that some adult patients may retain residual β-cell function sufficient to prevent ketoacidosis for many years although such individuals eventually become dependent on insulin for survival [11, 25, 26].

In this study 3-Screen negative samples were not tested in assays for individual autoantibodies and the possibility that some of these samples were positive for one or more individual autoantibodies cannot be excluded. Although this is a limitation of the study, the positivity for individual diabetes autoantibodies in 3-Screen negative patients is very rare [13]. Furthermore, all 44 patients found positive in 3-Screen were from one hospital which may be an additional limitation of this study. Consequently, a future study on a larger population would be helpful to confirm 3-Screen positivity among T2DM patients.

The association of autoimmune diabetes with other organ-specific autoimmune diseases is well known [27–31]. Our finding that 20 /44 (45%) 3-Screen positive patients were positive for at least one thyroid autoantibody and 4/20 (20%) of these were biochemically hypothyroid is consistent with the previous observations and suggests that islet cell autoantibody positive adult-onset diabetes patients are at risk of developing associated autoimmune conditions in particular, autoimmune thyroid disease. However, none of the 44 3-Screen positive patients were positive for the adrenal autoantibody 21-OHAb.

Overall, our studies indicate that the simple, sensitive and specific 3-Screen ICA™ ELISA is a convenient and cost-effective one-step strategy for screening of patients with adult onset T2DM in clinical practice and therefore has advantage compared to individual autoantibody assays. In particular, we have demonstrated that adult onset diabetes patients who are 3-Screen positive present a characteristic clinical phenotype associated with poor β-cell function and should be monitored more closely particularly with respect to their requirement for insulin. Identifying 3-Screen positivity in adult onset T2DM may lead to personalized medicine aimed at longer preservation of β-cell function, and ultimately, application of 3-Screen in clinical practice is expected to benefit the patients and reduce the burden on the health service.

## References

[1] Ahlqvist E, Storm P, Käräjämäki A (2018). Novel subgroups of adult-onset diabetes and their association with outcomes: a data-driven cluster analysis of six variables. Lancet Diabetes Endocrinology.

[2] Turner R, Stratton I, Horton V (1997). UKPDS 25: autoantibodies to islet-cell cytoplasm and glutamic acid decarboxylase for prediction of insulin requirement in type 2 diabetes: UK Prospective Diabetes Study Group. Lancet.

[3] Zinman B, Kahn SE, Haffner SM (2004). Phenotypic characteristics of GAD antibody-positive recently diagnosed patients with type 2 diabetes in North America and Europe. Diabetes.

[4] Davis TME, Wright AD, Mehta ZM (2005). Islet autoantibodies in clinically diagnosed type 2 diabetes: prevalence and relationship with metabolic control (UKPDS 70). Diabetologia.

[5] Zhou Z, Xiang Y, Ji L (2013). Frequency, immunogenetics, and clinical characteristics of latent autoimmune diabetes in China (LADA China Study). Diabetes.

[6] Wenzlau JM, Frisch LM, Gardner TJ (2009). Novel antigens in type 1 diabetes: the importance of ZnT8. Curr Diabet Rep.

[7] Lampasona V, Petrone A, Tiberti C (2010). Zinc transporter 8 antibodies complement GAD and IA-2 antibodies in the identification and characterization of adult-onset autoimmune diabetes. Diabet Care.

[8] Xiang Y, Huang G, Shan Z (2015). Glutamic acid decarboxylase autoantibodies are dominant but insufficient to identify most Chinese with adult-onset non-insulin requiring autoimmune diabetes: LADA China study 5. Acta Diabetol.

[9] Weng J, Zhou Z, Guo L et al (2018). Incidence of type 1 diabetes in China, 2010–13: population based study. Brit Med J 360: j5295. 10.1136/bmj.j5295 PMC5750780 29298776

[10] Sacks DB, Arnold M, Bakris GL (2011). Guidelines and recommendations for laboratory analysis in the diagnosis and management of diabetes mellitus. Diabetes Care.

[11] American Diabetes Association. 2 (2019). Classification and diagnosis of diabetes: standards of medical care in diabetes – 2019. Diabetes Care 42 (Suppl 1): S13-S28 10.2337/dc19-S002 30559228

[12] Diabetes Branch of Chinese Medical Association (2018). Guidelines for the prevention and treatment of type 2 diabetes in China (2017 edition). Chin J Diabetes Mellitus 10: 1.

[13] Amoroso M, Achenbach P, Powell M (2016). 3-Screen islet cell autoantibody ELISA: a sensitive and specific ELISA for the combined measurement of autoantibodies to GAD65, to IA-2 and to ZnT8. Clin Chim Acta.

[14] Brooking H, Ananieva-Jordanova R, Arnold C (2003). A sensitive non-isotopic assay for GAD_65_ autoantibodies. Clin Chim Acta.

[15] Chen S, Willis J, Maclean C (2005). Sensitive non-isotopic assays for autoantibodies to IA-2 and to a combination of both IA-2 and GAD_65_. Clin Chim Acta.

[16] Petruzelkova L, Ananieva-Jordanova R, Vcelakova,  (2014). The dynamic changes of zinc transporter 8 autoantibodies in Czech children from the onset of type 1 diabetes mellitus. Diabet Med.

[17] Bingley PJ, Bonifacio E, Mueller PW (2003). Diabetes antibody standardization program: first assay proficiency evaluation. Diabetes.

[18] Törn C, Mueller PW, Schlosser M (2008). Diabetes antibody standardization program: evaluation of assays for autoantibodies to glutamic acid decarboxylase and islet antigen-2. Diabetologia.

[19] Lampasona V, Schlosser M, Mueller PW (2011). Diabetes antibody standardization program: first proficiency evaluation of assays for autoantibodies to zinc transporter 8. Clin Chem.

[20] R Core Team (2021). R: A language and environment for statistical computing. R Foundation for Statistical Computing ,Vienna, Austria (https://www.R-project.org)

[21] Ziegler A-G, Kick K, Bonifacio E (2020). Yield of a public health screening of children for islet autoantibodies in Bavaria, Germany. J Am Med Assoc.

[22] Bottazzo GF, Bosi E, Cull CA (2005). IA-2 antibody prevalence and risk assessment of early insulin requirement in subjects presenting with type 2 diabetes (UKPDS 71). Diabetologia.

[23] Hawa MI, Kolb H, Schloot N (2013). Adult onset autoimmune diabetes in Europe is prevalent with a broad clinical phenotype: Action LADA 7. Diabetes Care.

[24] Kawasaki E, Okada A, Uchida A (2019). Discrepancy of glutamic acid decarboxylase 65 autoantibody results between RSR radioimmunoassay and enzyme-linked immunosorbent assay in patients with type 1 diabetes is related to autoantibody affinity. J Diabetes Investig.

[25] Palmer JP, Hirsch IB (2003). What's in a Name: Latent autoimmune diabetes of adults, type 1.5, adult-onset, and type 1 diabetes. Diabetes Care.

[26] Buzzetti R, Di Pietro S, Giaccari A (2007). High titer of autoantibodies to GAD identifies a specific phenotype of adult-onset autoimmune diabetes. Diabetes Care.

[27] Eisenbarth GS, Gottleib PA (2004). Autoimmune polyendocrine syndromes. New Engl J Med.

[28] Triolo TM, Armstrong TK, McFann K (2011). Additional autoimmune disease found in 33% of patients at type 1 diabetes onset. Diabetes Care.

[29] Shun CB, Donaghue KC, Phelan H (2014). Thyroid autoimmunity in type 1 diabetes: systematic review and meta-analysis. Diabet Med.

[30] Volzke H, Krohn U, Wallaschofski H (2007). The spectrum of thyroid disorders in adult type 1 diabetes mellitus. Diabetes Metab Res Rev.

[31] Fleiner HF, Bjoro T, Midthjell K (2016). Prevalence of thyroid dysfunction in autoimmune and type 2 diabetes: the population-based HUNT study in Norway. J Clin Endocrinol Metab.

